# Host and Viral Genetic Variation in HBV-Related Hepatocellular Carcinoma

**DOI:** 10.3389/fgene.2018.00261

**Published:** 2018-07-19

**Authors:** Ping An, Jinghang Xu, Yanyan Yu, Cheryl A. Winkler

**Affiliations:** ^1^Basic Research Laboratory, National Cancer Institute, Leidos Biomedical Research, Inc., Frederick National Laboratory for Cancer Research, Frederick, MD, United States; ^2^Department of Infectious Diseases, Center for Liver Diseases, Peking University First Hospital, Peking University, Beijing, China

**Keywords:** genotype, hepatitis B virus, hepatocellular carcinoma, mutation, single nucleotide polymorphisms

## Abstract

Hepatocellular carcinoma (HCC) is the fifth most common cancer in men and the second leading cause of cancer deaths globally. The high prevalence of HCC is due in part to the high prevalence of chronic HBV infection and the high mortality rate is due to the lack of biomarkers for early detection and limited treatment options for late stage HCC. The observed individual variance in development of HCC is attributable to differences in HBV genotype and mutations, host predisposing germline genetic variations, the acquisition of tumor-specific somatic mutations, as well as environmental factors. HBV genotype C and mutations in the *preS, basic core promoter* (*BCP*) or *HBx* regions are associated with an increased risk of HCC. Genome-wide association studies have identified common polymorphisms in *KIF1B, HLA-DQ, STAT4*, and *GRIK1* with altered risk of HBV-related HCC. HBV integration into growth control genes (such as *TERT*), pro-oncogenic genes, or tumor suppressor genes and the oncogenic activity of truncated HBx promote hepatocarcinogenesis. Somatic mutations in the *TERT promoter* and classic cancer signaling pathways, including Wnt (*CTNNB1*), cell cycle regulation (*TP53*), and epigenetic modification (*ARID2* and *MLL4*) are frequently detected in hepatic tumor tissues. The identification of HBV and host variation associated with tumor initiation and progression has clinical utility for improving early diagnosis and prognosis; whereas the identification of somatic mutations driving tumorigenesis hold promise to inform precision treatment for HCC patients.

## Introduction

Hepatocellular carcinoma (HCC) is the fifth most common cancer in men and the second leading cause of cancer deaths worldwide (El-Serag, 2011; Torre et al., 2015). HCC prevalence is highest in East and Southeast Asia and sub-Saharan Africa, but the incidence rates of HCC have increased in the United States and Western Europe over the past few decades (Lee, 2015; Njei et al., 2015). Early diagnosis and surgical resection remain the key to potential curative treatment; however, most HCC patients present with late stage tumors and have poor prognosis. HCC surveillance is mainly based on sonography and alpha-fetoprotein (AFP) measurement, both of which lack sufficient sensitivity and specificity (Yim and Lok, 2006; Bruix et al., 2011).

Hepatitis B virus (HBV) is one of the leading risk factors for HCC, especially in HBV endemic areas. Despite the existence of an effective vaccine, about 257 million people, or 3.5% of the global population, were afflicted with chronic HBV infection in 2015, WHO (2017). The clinical spectrum of chronic HBV infection ranges from asymptomatic carrier status to chronic hepatitis B (CHB), which may evolve to liver cirrhosis and HCC (EASL, 2012). An estimated 8–20% of untreated adults with CHB will develop liver cirrhosis within 5 years (Terrault et al., 2016) and 2–8% of those with cirrhosis develop HCC annually (Yim and Lok, 2006; Bruix et al., 2011; Hung et al., 2017).

Environmental and host risk factors for HCC include childhood acquisition of HBV infection, cirrhosis, aflatoxin B1 exposure, heavy alcohol use, smoking, male sex, advancing age, obesity, type 2 diabetes and an impaired immune response (Bruix et al., 2016). In addition to HBV, hepatitis C virus (HCV) chronic infection is a major cause of HCC, but co-infection by HBV and HCV do not seem to confer greater HCC risk than HBV or HCV monoinfection in a meta-analysis (Cho et al., 2011). Co-infection of HBV and hepatitis delta virus (HDV), occurring in ~5% of those HBV infected, was associated with 3 to 6-fold higher incidence of HCC in longitudinal cohorts (Fattovich et al., 2000; Romeo et al., 2009; Ji et al., 2012; Kushner et al., 2015).

Family clustering and incidence differences among different ancestry groups suggests that inherited genetic factors may contribute to HCC risk (Shen et al., 1991). Common and rare single nucleotide polymorphisms (SNPs) and structural genomic changes may predispose or restrict HCC development. The identification of moderate to high penetrant genetic variants associated with HBV-related HCC might identify HCC susceptible patients that would lead to earlier intervention and better outcomes (Thomas et al., 2009; Brennan et al., 2011; Michailidou et al., 2015). For example, screening for *BRCA1* and *BRCA2* mutations is routinely used to identify women at risk for breast and ovarian cancer and is routinely used in precision treatment protocols (Brennan et al., 2011; Michailidou et al., 2015).

Somatic mutations occurring in hepatocytes as the consequence of exogenous and endogenous mutagenic factors, are likely involved in HCC initiation and progression, as they are in many other cancers. Identification of cancer diver mutations in HCC are essential for HCC prognostics and for precision therapy targeting perturbed pathways.

Here, we review recent advancements and identify knowledge gaps in HBV-related HCC genetics at three levels: the human host, the virus, and somatic mutations in liver tumors and their associations with clinical outcomes.

## HBV

**Figure 1:**
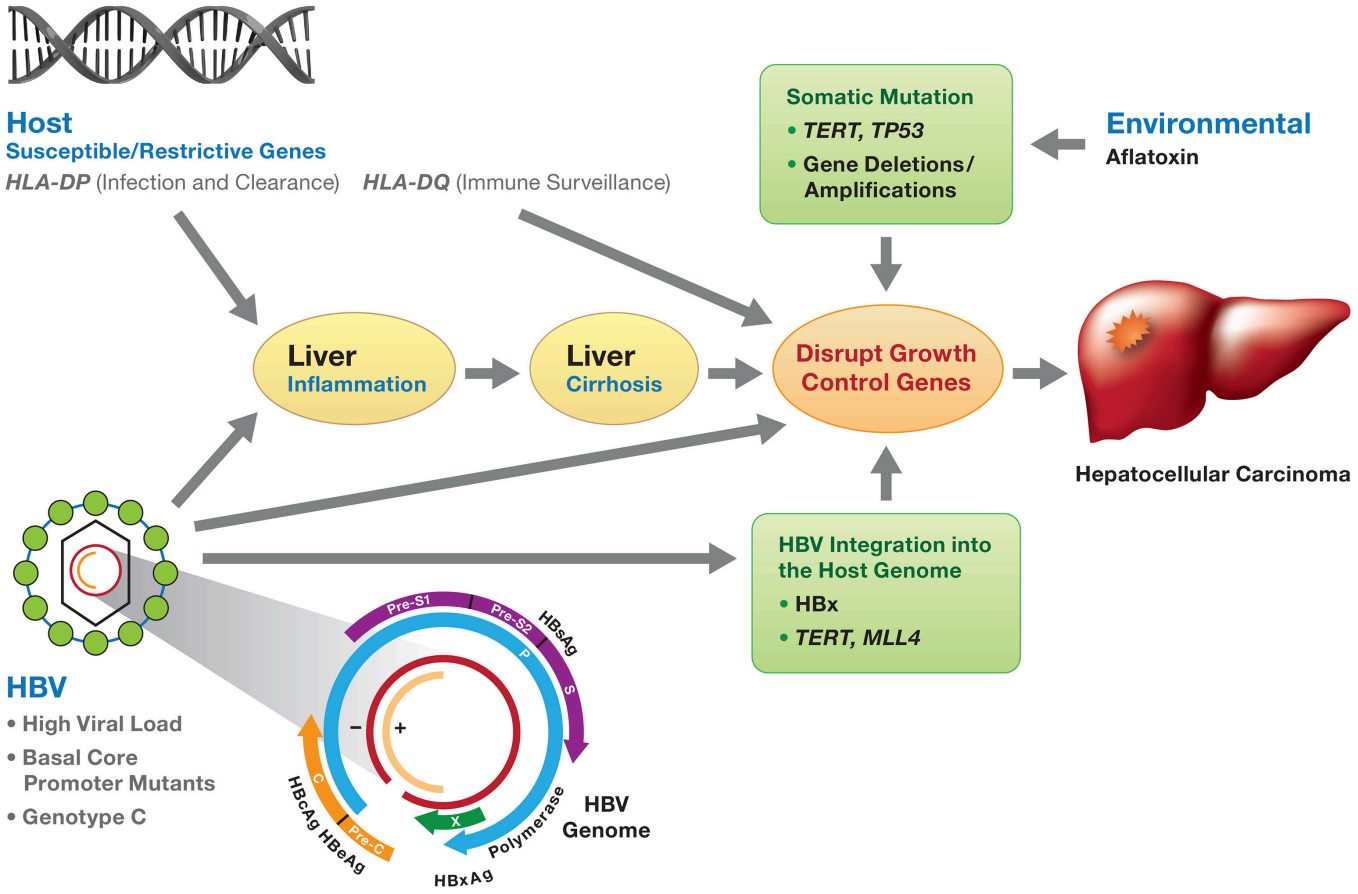
Viral and host genetic factors involved in the development of HBV-induced HCC. HBV infection, if not resolved, may develop to chronic hepatitis and progress to liver cirrhosis and subsequently HCC. Molecular mechanisms of HBV-related HCC involve (1) chronic inflammation and regeneration of hepatocytes; (2) accumulation of genetic alterations that confer cell growth advantage; (3) integration of HBV DNA into the host genome and activation of host genes controlling cell proliferation; (4) genomic instability; and (5) direct promotion of cell proliferation by viral proteins (mainly HBx). The development of HCC is the consequence of the interaction of environmental factors (e.g., aflatoxin), HBV viral factors (genotypes, HBV DNA levels and HBV mutants) and host genetic susceptible variants (e.g., *HLA-DP* variants affecting HBV clearance), along with somatic mutations (*TP53, TERT*, gene deletion/amplification) disrupting cell growth control. HBV genotype C and mutations in the *preS, basic core promoter* or *HBx* gene are associated with an increased risk of HCC.

HBV has a partially double-stranded circular DNA genome of ~3.2 kilobase (kb) pairs, comprising four overlapping open reading frames (ORF): *preS/S, P, preC/C*, and *X* (Liang, 2009). The *PreS/S* gene region encodes large (preS1+preS2 +S), middle (preS2+S), and small (S) HBsAg envelope proteins (Figure 1). The *P* region encodes the polymerase/reverse transcriptase, which is involved in genome replication. The preC/C codes the nucleocapsid hepatitis B core antigen (HBcAg) or the hepatitis B e antigen (HBeAg) translated from initiated codons at the core or precore regions, respectively. HBcAg and HBeAg are biomarkers for HBV active infection or infectivity. The *X* ORF encodes a nonstructural protein (HBx) with multiple functions in viral replication and oncogenic activity (Liang, 2009).

### Molecular mechanisms of HBV-related HCC

There are at least three prevailing mechanisms proposed for the development of HBV-related HCC (Kremsdorf et al., 2006; Block et al., 2007; Hai et al., 2014; Figure 1). First, chronic inflammation and regeneration of hepatocytes during chronic HBV infection may lead to the accumulation of genetic alterations that confer cell growth advantage. Second, the integration of HBV DNA into the host genome may activate the host genes controlling cell proliferation and cause genomic instability. Finally, HBV proteins, mainly HBx, may promote cell proliferation (Kremsdorf et al., 2006; Block et al., 2007; Hai et al., 2014). It is also likely that all three mechanisms contribute to HCC development.

### HBV viral load

The general consensus is that persistent high-level HBV replication poses greater risk of developing liver cirrhosis and HCC (Sanchez-Tapias et al., 2002; Chen et al., 2006a,b, 2007; Fattovich et al., 2008). A large prospective study, which followed 3653 HBsAg positive participants enrolled in the Taiwanese Reveal-HBV cohort for over a decade, found that HBV DNA levels at study entry were positively correlated with incidence of HCC in a dose-dependent manner. Individuals with HBV levels greater than 1 million copies /mL were 10-fold more likely to develop HCC than those with less than 300 copies/mL (Chen et al., 2006b). Serum HBV DNA viral load is also associated HCC tumor recurrence (Hung et al., 2008; Wu et al., 2009). A viral load of greater than 10,000 copies/mL (2000 IU/mL) was independently associated with HBV-related HCC recurrence in patients who underwent liver resection (Hung et al., 2008). Antiviral therapy in these patients decreased tumor recurrence (Li et al., 2010).

### HBV genotype

Ten HBV genotypes (A to J) that diverge by >8% of their nucleotide sequences have been identified globally. HBV genotypes distribute within distinct geographic regions and ethnic populations(Lin and Kao, 2015). Phylogenetic analysis of HBV genotypes indicate that global distribution of HBV genotypes corresponds with the major prehistoric and modern human migration patterns, after HBV established infection in humans around 33,000 years ago (Paraskevis et al., 2013). Different genotypes prevail in the two regions with the highest HBV and HCC prevalence: genotypes B and C are prevalent in East Asia where vertical transmission is predominant, whereas genotypes A and D are prevalent in sub-Saharan Africa where horizontal transmission is more common. HBV genotypes A and D are the main genotypes in low HBV prevalence regions including Europe and North America (Lin and Kao, 2015).

The risk of HCC appears to differ by HBV subtypes. In East Asia, HBV genotype C is associated with higher risk of HCC compared to genotype B (Chan et al., 2004; Yu et al., 2005; Yang et al., 2008; Tseng et al., 2012; Vutien et al., 2013; Lin and Kao, 2015; Raffetti et al., 2016). Serum HBV viral load is significantly higher in patients with HBV genotype C than in those infected with genotype B (Chu et al., 2002; Chan et al., 2004; Yu et al., 2005; Lin and Kao, 2015). The increased risk of HCC might be the consequence of longer exposure to high levels of HBV in patients with genotype C (Kao et al., 2004; Ni et al., 2004). In contrast, HCC risk for genotype D and A appear to be similar, although there are reports that patients with HBV genotype A experience longer sustained remissions (Sanchez-Tapias et al., 2002) and are more responsive to interferon alpha (IFN- α) treatment compared to those infected with HBV genotype D (Erhardt et al., 2005). There are at least 5 co-circulating HBV genotypes in Alaska Native people, which offers rare opportunity for direct comparisons of HCC risk among genotypes. A longitudinal cohort of Alaska Natives followed for over 30 years reported that carriers of genotypes A (OR 4), C (OR 16), and F (OR 14) are at higher risk compared with genotypes B or D (Livingston et al., 2007; Ching et al., 2016). A study of 1,000 Alaska Native children and young adults followed for over 23 years found that HBV genotype F1 was associated with the highest risk for HCC (Gounder et al., 2016). These studies support a strong role for HBV genotypes in predicting HCC risk. In comparison, HCV genotype 3 was also associated with higher risk of HCC compared to other HCV genotypes (van der Meer et al., 2012; Kanwal et al., 2014).

### HBV mutations

HBV DNA replication errors occur at a much higher rate than for other DNA viruses because HBV reverse transcriptase lacks a proofreading function (Chotiyaputta and Lok, 2009). The estimated nucleotide substitution rate was 2.2 × 10^−6^ substitutions/site/year, which is higher than other DNA viruses but lower than RNA viruses (Paraskevis et al., 2013). Mutations in the HBV genome commonly associated with HCC are summarized in Table 1, with the *preC/C* and basic core promoter (*BCP*) regions harboring the most frequent mutations.

**Table 1:** The association of HBV mutants with HCC risk.

**Mutation**	**Frequency^s^**		**RR or OR (95% CI)**	**References**
	**HCC**	**non-HCC**		
***PreS/S*** **mutants**				
Any *preS* mutation	45.5-72.3%	18.1-26.0%	3.82 (2.59-5.61)^b^	Liu et al., 2009; Yang et al., 2015
*PreS* deletion	29.2-51.1%	11.3-18.2%	3.98 (2.28-6.95)^b^	Liu et al., 2009; Yang et al., 2015
*PreS2* start codon mutation	17.7-21.2%	7.2-8.0%	2.63(1.30-5.34)^b^	Yang et al., 2015
***P*** **mutants**				
rtA181T/sW172*	Changes over the treatment duration of NAs.		Cumulative incidence of HCC: 1.8% (2/113) in the mutation-absent; 30% (3/10) in the mutation-present	Yeh et al., 2011
***PreC/C*** **mutants**				
BCP, A1762T/G1764A (xK130M/V131I)	66.5%(2,480/3,729)	39.8% (2,594/6,511)	3.05 (2.35-3.95)	Yang Z. et al., 2016
Enhancer II, C1653T	26.7-46.2%	13.3-28.2%	1.83 (1.21-2.76)^b^	Liu et al., 2009; Yang et al., 2015
BCP, T1753V (xI127T)	25.9-46.2%	15.5-23.5%	2.09 (1.49-2.94)^b^	Liu et al., 2009; Yang et al., 2015
*PreC*, G1896A	38.8-84.6%	27.9-82.7%	0.77 (0.47-1.26)^b^	Liu et al., 2009; Yang et al., 2015
***X*** **mutants**				
A10R/S144R double mutation	Not reported.			Shi et al., 2016
xK130M+xV131I+ xV5M (triple) mutations	41.9% (13/31)	11.9%(5/42)	5.34 (1.65-17.309)	Lee et al., 2011

*^a^ Frequency ranges were extracted from studies included in the meta-analyses (Liu et al., 2009; Yang et al., 2015)*.

b *Pooled RR from reference Yang et al. (2015)*.

*CI, confidence interval; NAs, nucleos(t)ide analogs; OR, odds ratio; RR, relative risk*.

### Pres/S region mutants

*PreS* mutations, especially the *preS1* deletion, *preS2* deletion, and *preS2* start codon mutations, may induce an unbalanced production of envelope proteins that accumulate in the endoplasmic reticulum (ER) of the hepatocytes, leading to ER stress, oxidative DNA damage and genomic instability (Pollicino et al., 2014). These cytotoxic effects cause liver cell damage and regeneration (Pollicino et al., 2014). A meta-analysis of 9 cross-sectional studies including 388 HCC cases demonstrated that any one of the *preS* mutants (C1653T, T1753V, and A1762T/G1764A) was associated with a 3.8-fold increased risk of HCC (Liu et al., 2009), consistent with the result from a recent meta-analysis of prospective studies comprising 360 cases (RR 3.8) (Yang et al., 2015). *PreS/S* deletions and the *Pre S2* start codon mutation had a RR 4.0 and 2.6, respectively (Yang et al., 2015). Specifically, C2964A, C3116T, and C7A were independently associated with an increased risk of HCC (Yin et al., 2010).

Mutations introducing stop codons in the S genomic region have been proposed to enhance tumor development by encoding truncated proteins with transcriptional transactivation activity (Pollicino et al., 2014). The premature stop codon mutations at position 172 or 182 in the S genomic region are associated with higher risk for liver cirrhosis and HCC (Lai et al., 2009; Pollicino et al., 2014).

### Prec/C region mutants

Mounting evidence supports the importance of the naturally occurring *basal core promoter (BCP)* double mutation, A1762T/G1764A, as a risk factor for HCC (Fang et al., 2008; Yang et al., 2008, 2015; Yang Z. et al., 2016). In a meta-analysis of 15 prospective studies comprising 1336 HCC cases, individuals with the double mutation had a 3-fold higher risk of developing HCC (Yang et al., 2015). In a more recent meta-analysis that included 3729 HCC cases, double mutation carriers had a 5-fold higher HCC risk (Yang Z. et al., 2016). Other prospective long-term follow-up studies found that the incidence of HCC in double mutation carriers was 4 to 5-fold higher (Fang et al., 2008; Yang et al., 2008). The presence of the *BCP* double mutation reduces HBeAg production and viral load before anti-HBe seroconversion (Buckwold et al., 1996; Fang et al., 2009), suggesting that its oncogenic mechanism is not directly attributable to viral replication level. However, the *preC* stop codon G1896A, which prevents the production of HBeAg, does not appear to affect HCC risk as demonstrated in a recent meta-analysis with 600 HCC cases (Yang et al., 2015).

### Polymerase/reverse transcriptase region mutants

Anti-HBV nucleos(t)ide analogs (NAs) are effective in lowering HBV DNA levels through their inhibition of HBV polymerase/reverse transcriptase (Terrault et al., 2016). However, the emergence of and the selection for resistance mutations in the polymerase/reverse transcriptase region confer a virial survival advantage and is a major barrier to the success of NAs treatment. In a longitudinal study of lamivudine (LMV)-resistant CHB patients, emergence of the rtA181T/sW172X mutant in LMV-resistant patients increased the risk of HCC (Lai et al., 2009; Yeh et al., 2011). NIH3T3 cells expressing this mutant showed greater oncogenic potential in nude mice (Lai and Yeh, 2008; Lai et al., 2009). It is an open question whether resistance to other nucelos(t)ide analogs also increases the risk of HCC.

### X region mutants

HBx protein is a transcriptional activator of various host cellular genes involved in growth control, DNA repair, and epigenetic modification (Xu et al., 2014). It activates the Ras/Raf/mitogen-activated protein (MAP) kinase pathway, which is involved in hepatocarcinogenesis, and interacts with the tumor protein 53 gene (*TP53*), interfering with its function as a tumor suppressor (Di Bisceglie, 2009).

Certain point mutations in the *HBx* gene, in particular, the K130M and V131I double substitutions, are more frequent in patients with liver cirrhosis and/or HCC than in patients with chronic hepatitis B (Baptista et al., 1999). Due to the partial overlap of BCP with Hbx, the same nucleotide substitutions result in both HBx K130M and V131I and pre-core *BCP* A1762T/G1764A changes. Increasing number of *HBx* mutations is correlated with increased risk of HCC. The double xK130M+xV131I and triple xK130M+xV131I+ xV5M mutations are associated with a 4-5-fold increased the risk of HCC (Lee et al., 2011). Substitutions at position 10, 30, 38, 88, 94, and 144 have also been reported to be associated with HCC (Muroyama et al., 2006; Lee et al., 2011; Wang et al., 2012; Shi et al., 2016; Table 1). Even though the precise mechanism remains to be elucidated, *HBx* plays a central role in hepatocarcinogenesis and could be an attractive therapeutic target for HCC suppression.

In summary, major HBV viral factors associated with increased risk of HCC include genotype C and F, higher HBV-DNA levels, mutations in the *preS/S region*, the double mutation A1762T/G1764A in the basic core promoter, and the double or triple mutation K130M, V131I xV5M in the *HBx* gene. Different genotypes of the virus (which are due to the accumulation of viral mutations over time) have differing propensities for the development of HCC. Epidemiological association data and some functional evidence suggest the likely causal role of viral mutations, particularly for genotype differences. However, the temporal and causal relationship of HBV mutations with HCC remains to be firmly established, as most results were obtained from case-control and cross-sectional studies and rarely from prospective longitudinal HBV cohorts. HBV mutations may also increase risk of HCC indirectly by the acquisition of mutations that leads to immune escape and increased viral replication. Higher HBV burden would contribute to chronic inflammation promoting the destruction and regeneration of HBV infected hepatocytes mediated by host cytotoxic T lymphocyte (CTL) immune response and increasing the opportunity for replication errors during cell mitosis (Block et al., 2007). The emergence and accumulation of genetic and epigenetic alterations with cell growth advantage eventually lead to hepatocarcinogenesis (Block et al., 2007). The exact molecular mechanisms by which HBV mutations promote hepatocarcinogenesis warrants further investigation.

## Host genetic variation and HBV-related HCC

Although the incidence of HCC among HBV carriers is much higher (223-fold) than among non-carriers (Beasley et al., 1981), only a fraction of patients with chronic HBV infection develop HCC. Results from twin studies, family clustering studies and incidence differences between continental ancestry groups suggest that host genetic factors contribute to HCC susceptibility (Shen et al., 1991). Genome-wide association studies (GWAS) provide an agnostic (hypothesis-free) method to identify susceptibility and resistance loci for HBV-related HCC. The ability to securely identify genetic susceptibility loci associated with HCC is critically dependent on sample size for sufficient power to detect associations with small to moderate effect sizes in GWAS. This is due to the need to correct for multiple testing that tends to inflate false positive signals; therefore, the human genetics community has determined that a genome-wide significance threshold of *p* < 5 × 10^−8^ and independent replication association is required to firmly establish a genotype-phenotype association. The top associations between host genetic variants with HCC are summarized in Table 2. Only associations in the HLA class II region have been found to replicate in at least two studies. These studies, performed primarily in East Asians, suggest that no common, moderate to high penetrant alleles contribute to HCC development.

**Table 2:** Associations of host genetic variants with HBV-related HCC from GWASs and replication studies.

**Chr**.	**Nearest gene**	**SNP**	**Location**	**Major/ minor allele**	**Population**	**Case**	**Control**	**No. cases**	**No. control**	**MAF cases**	**MAF controls**	**Model**	**OR**	**95% CI**	***P*-value**	**Study design**	**References**
1p36.22	*KIF1B*	rs17401966	Intron	A/G	Chinese	HBV-HCC	HBV carriers	2,310 (discovery 348)	1,789	0.19	0.28	Add	0.62	0.56–0.69	3.4 × 10^−19^	GWAS+ 4 replications	Zhang et al., 2010
					Han Chinese	HBV-HCC	HBV carriers	1,293	1,334	0.27	0.28	Additive	0.99	0.88–1.12	0.92	Replication	Hu et al., 2012
					Japanese, Korean, Hong Kong Chinese	HBV-HCC	HBV carriers ASC+CHB	578	1,351	0.26	0.24	Allelic	1.00	0.85–1.19	0.97	4 replications	Sawai et al., 2012
					Thai	HBV-HCC	HBV carriers	202	196	0.30	0.30	Allelic	0.95	0.70–1.28	0.733	Replication	Sopipong et al., 2013
					Chinese	HBV-HCC	HBV carriers	1,161	1,353	0.27	0.28	Additive	0.98	0.87–1.11	0.79	GWAS	Jiang et al., 2013
					Southern Chinese	HBV-HCC	Non-HCC HBV carriers	357	354	0.29	0.29	Allelic	1.0	NA	0.91	Replication	Chan et al., 2011
					Han Chinese	HBV-HCC	HBV carrier	1,538	1,465	0.27	0.29	Additive	0.90	0.80–1.02	0.091	GWAS	Li et al., 2012
2q32.2-2q32.3	*STAT4*	rs7574865	Intron	G/T	Chinese	HBV-HCC	Non-HCC HBV carriers	5,480 (discovery 1,161)	6,319	0.28	0.32	Additive	0.83	0.78–0.88	2.48 × 10^−10^	GWAS+ 6 replications	Jiang et al., 2013
6p21.32	*HLA- DQA2/DQB1*	rs9275319	Intergenic	A/G	Chinese	HBV-HCC	Non-HCC HBV carriers	5,480 (discovery 1,161)	6,319	0.08	0.11	Additive	0.67	0.61–0.74	2.72 × 10^−17^	GWAS+ 6 replications	Jiang et al., 2013
6p21.32	*HLA-DQA1/DRB1*	rs9272105	Intergenic	G/A	Han Chinese	HBV-HCC	HBV carrier	5,885 (discovery 1,538)	6,181	0.51	0.45	Additive	1.28	1.22–1.35	5.24 × 10^−22^	GWAS+ 2 validations + 1 replication	Li et al., 2012
21q21.3	*GRIK1*	rs455804	Intron	C/A	Han Chinese	HBV-HCC	HBV carrier	5,967 (discovery 1,538)	6,182	0.3	0.34	Additive	0.84	0.80–0.89	5.24 × 10^−10^	GWAS+ 2 validations + 1 replication	Li et al., 2012

*ASC, asymptomatic carrier; CHB, chronic hepatitis B; Chr., chromosome; CI, confidence interval; GWAS, Genome-Wide Association Study; HBV, hepatitis B virus; HCC, hepatocellular carcinoma; MAF, Minor allele frequency; OR, odds ratio*.

The HCC association of intergenic SNPs near *HLA-DQA1* and *HLA-DQA2*, and *HLA-DQB1*, encoding the alpha and beta chains of the HLA-DQ protein, were identified by GWAS in two large studies (Li et al., 2012; Jiang et al., 2013). HLA class II proteins, comprising HLA-DP, DQ, and DR proteins, play a central role in extracellular antigen presentation to CD4 T cells stimulating B cell that lead to antibody production against foreign pathogens, including HBV and HCV.

Outside of the HLA region, genetic associations with HBV-related HCC have been reported for several genes. A two-stage GWAS composed of five cohorts from East Asian identified the intronic rs17401966 SNP in the *KIF1B* gene (OR = 0.61) with genome-wide significance in a meta-analysis (Zhang et al., 2010). *KIF1B* encodes a kinesin superfamily member involved in the transport of organelles and vesicles. Both germline and somatic loss-of-function mutations in the KIF1B isoform have been detected in multiple cancers (Zhang et al., 2010). However, this association failed to replicate in multiple independent studies, several of which were well-powered (Table 2; Hu et al., 2012; Li et al., 2012; Sawai et al., 2012; Jiang et al., 2013; Sopipong et al., 2013). The latest meta-analysis of 12 cohorts found statistical significance only when the original 5 cohorts reporting positive associations were included, but when these 5 cohorts were removed the association was abrogated (Su et al., 2017). Further validation and functional studies are warranted to support or not a role of *KIF1b* variation in HCC.

Other candidate genes implicated by GWAS include *STAT4*, and *GRIK1* (Table 2; Li et al., 2012; Jiang et al., 2013). STAT4 is a transcription factor involved in development of Th1 cells and production of IFN-γ, a cytokine with antiviral and antitumor activities. STAT4 rs7574865 is an expression quantitative trait locus (eQTL) with dosage effect in HCC tissues (Jiang et al., 2013). Glutamate receptor GRIK1, is involved in cancer development (Li et al., 2012), though its functional role in HCC has not been experimentally demonstrated. These associations have not yet been replicated or validated by functional assessment. Recently, a functional genomic approach revealed a c-Myc binding SNP that regulates a putative tumor suppressor gene EPB41 and was associated with predisposition to HCC (Yang et al., 2016b).

A 2011 experts′ commentary highlighted the need for further GWAS studies of HCC with larger subject enrollment, clearly delineated phenotypes, and replication (Budhu and Wang, 2011). The lack of replication for HCC GWAS studies is most likely due to relatively small sample sizes of the study although genetic and phenotypic heterogeneity may have also contributed. True causal variants should have similar effects across all populations while marker SNPs may have different effects in different ancestry or ethnic populations. These differences in effect size or statistical significance in haplotype structure among different populations, the inclusion of trans-ethnic or continental racial populations can be used to refine position of the causal locus by fine-mapping (Franceschini et al., 2012). The newer population specific genotyping arrays, in combination with larger case-control groups representing diverse populations, should reveal additional germline variants that contribute to the observed variance in HBV-HCC susceptibility. Finally, whole genome or exome sequencing might also identify causal rare variants associated with HBV-HCC. These studies should identify critical pathophysiological pathways for HBV-related liver cirrhosis and HCC.

## Interaction of host genes and HBV: HBV DNA integration, somatic mutations and epigenetic modification in HCC oncogenesis

Environmental factors, HBV genotypes and genetic variation, and host genetic variation are involved in the development and progression of HCC. The interplay between these factors might lead to the initiation of HCC (Figure 1).

### HBV integration and HCC

HBV DNA integration into the host genome is one of the proposed molecular mechanisms of hepatocarcinogenesis. HBV-DNA integration occurs during both the acute and chronic stages and integrated HBV-DNA is detectable in 75-90% of HCC tissues (Murakami et al., 2005; Zhao et al., 2016). HBV integration events may cause direct gene disruption, HBV promoter-driven transcription of host genes, viral-host transcript fusion and induce genome instability (Tao et al., 2011; Jiang et al., 2012; Sung et al., 2012), which may lead to activation of proto-oncogenes or inactivation of tumor suppressor genes. Oncogenic activity of the cellular and viral genes resulting from the HBV integration confers a selective growth advantage to cells with accumulation of genetic defects, leading to hepatocarcinogenesis (Tao et al., 2011; Jiang et al., 2012; Sung et al., 2012).

Advanced genome-wide sequencing technology has enabled non-biased genome scans of HBV integration sites in HCCs (Table 3; Murakami et al., 2005; Ding et al., 2012; Toh et al., 2013; Lau et al., 2014). HBV integration sites are randomly distributed across the whole genome with a handful of hotspots (Sung et al., 2012). Some investigators found preferential integration in chromosome 10 and 17 (Ding et al., 2012; Toh et al., 2013; Zhao et al., 2016). HBV appears to preferentially integrate near coding genes that are transcriptionally active (Ding et al., 2012; Sung et al., 2012; Toh et al., 2013), as was observed for HIV integration (Maldarelli et al., 2014).

**Table 3:** HBV integration into host genes in HCC tissues from next generation sequencing studies.

**Frequency**	**Host gene**	**References**
Common	*TERT*	Paterlini-Brechot et al., 2003; Murakami et al., 2005; Ding et al., 2012; Fujimoto et al., 2012; Sung et al., 2012; Li et al., 2013; Toh et al., 2013; Lau et al., 2014; Kawai-Kitahata et al., 2016
Less Common	*MLL4, CCNE1*	Saigo et al., 2008; Ding et al., 2012; Jiang et al., 2012; Sung et al., 2012; Li et al., 2013
Infrequent	*SMAD5, SENP5, ROCK1, PDGFRB, MAPK1, ANGPT1, CYP2C8, CCNA2, MSMB, AIP, NTN1, FAS, CCNG1, P62*	Murakami et al., 2005; Tao et al., 2011; Ding et al., 2012; Jiang et al., 2012; Sung et al., 2012; Toh et al., 2013
	**Viral gene**
Common	*ct-HBx*, HBx-LINE1 RNA hybrid	Yin et al., 2013; Lau et al., 2014; Wang et al., 2014; Zhu et al., 2015; Li et al., 2016
	**Chromosome**
Common	Chromosome 10, 17, 8p11	Murakami et al., 2005; Ding et al., 2012; Toh et al., 2013; Lau et al., 2014

Recurrent HBV integration sites have been detected in many cancer-related genes. The highest frequency of HBV integration is detected in the telomerase reverse transcriptase (*TERT*) gene (Paterlini-Brechot et al., 2003; Murakami et al., 2005; Ding et al., 2012; Fujimoto et al., 2012; Sung et al., 2012; Li et al., 2013; Toh et al., 2013; Lau et al., 2014; Kawai-Kitahata et al., 2016). Integration in the proximity of TERT is correlated with *TERT* gene expression (Sung et al., 2012; Zhao et al., 2016); reactivation of TERT likely confers early clonal advantage during chronic HBV infection. Whole-genome sequencing identified clonal expansion of HBV integration in the *TERT* locus in HCC tumors but not in adjacent non-tumor tissue DNA, suggesting its role in liver carcinogenesis (Fujimoto et al., 2012). Additional commonly identified recurrent target genes for HBV integration in HCC liver tissue are listed in Table 3, including *MLL4* and *CCNE1* (Saigo et al., 2008; Ding et al., 2012; Jiang et al., 2012; Sung et al., 2012; Li et al., 2013) among others (Murakami et al., 2005; Tao et al., 2011; Ding et al., 2012; Jiang et al., 2012; Sung et al., 2012; Toh et al., 2013). Individuals with a high number of integration sites have unfavorable HCC survival (Murakami et al., 2005; Ding et al., 2012; Toh et al., 2013; Lau et al., 2014; Zhao et al., 2016).

The integrated HBV genome itself may be oncogenic (Ding et al., 2012; Fujimoto et al., 2012; Jiang et al., 2012; Li et al., 2013; Toh et al., 2013). Upon integration, the 3′-end of the HBx is often deleted, resulting in C-terminal truncated HBx (ct-HBx) protein, which contributes to HCC initiation and progression (Tu et al., 2001; Ma et al., 2008; Sze et al., 2013; Yin et al., 2013; Wang et al., 2014; Zhu et al., 2015; Li et al., 2016). The truncated HBx protein is reported to promote the transforming ability of hepatocytes (Tu et al., 2001), induce C-Jun/MMP10 activation to increase cellular proliferation (Ma et al., 2008; Sze et al., 2013), as well as enhance hepatoma cell invasion and metastasis (Sze et al., 2013; Li et al., 2016). Ct- HBx is present significantly more in tumors compared to adjacent non-tumorous tissues in several studies (Yin et al., 2013; Wang et al., 2014; Zhu et al., 2015; Li et al., 2016) and Ct-HBx expression in HCC tissue correlates with decreased patient survival (Yin et al., 2013).

Quantification of HBV integration sites may have prognostic value in predicting HCC survival. In a genome-wide survey of 88 HCC patients, increasing number of HBV integrations was correlated with HCC occurrence at younger age and shorter survival (Sung et al., 2012). A viral-human chimeric HBx-LINE1 RNA hybrid, detected in 23% of HCC tumors, was also predictive of poorer survival (Lau et al., 2014).

### Somatic mutations and copy number alteration in HCC

Most somatic mutations are harmless passenger mutations accumulated in the process of tumor growth, which occur at random and confer no selective advantage for tumor cells. Driver mutations confer selective growth advantage and cause transformation of a normal cell to a cancer cell, which are clinically relevant. Recurrent coding changes in multiple tumor cases may identify putative cancer driver mutations (Bozic et al., 2010). Recent whole-genome and whole-exome deep sequencing of tumor and non-tumor tissues have revealed the importance of somatic mutations and structural variations in tumor development across cancer types and has led to more efficacious treatment modalities based on molecular changes or genomic classification rather than target organs of cancer (e.g., *BRAF* V600E, *KIT, EGFR, ERBB2*) (Swanton et al., 2016).

Recurrent somatic mutations in HCC have been identified in several known cancer-related genes and pathways. These include genes required for telomere maintenance, the Wnt signaling pathway, which regulates cell proliferation, cell cycle regulation, epigenetic modification, and the PI3K/Akt/mTOR, Ras/Raf/MAP, oxidative stress, and JAK/STAT pathways (Li et al., 2011; Woo et al., 2011; Fujimoto et al., 2012; Guichard et al., 2012; Huang et al., 2012; Cleary et al., 2013; Kan et al., 2013; Nault et al., 2013; Ahn et al., 2014; Meng et al., 2014; Totoki et al., 2014; Schulze et al., 2015; Kawai-Kitahata et al., 2016; Yao S. et al., 2016). The perturbed genes identified in HCC tumor genomes are listed in Table 4. Copy number alterations result from chromosomal focal amplifications of some oncogenes and less frequently from homozygous deletions of tumor suppressors (Sawey et al., 2011; Guichard et al., 2012; Wang et al., 2013; Totoki et al., 2014; Schulze et al., 2015). Recurrent focal amplifications have been observed for *TERT, MET*, and others (Sawey et al., 2011; Guichard et al., 2012; Wang et al., 2013; Totoki et al., 2014; Schulze et al., 2015), while recurrent homozygous deletions have been reported for *CDKN2A, ARID1A*, and others (Table 4; Guichard et al., 2012; Wang et al., 2013; Totoki et al., 2014; Schulze et al., 2015).

**Table 4:** Recurrent somatic mutations in HCC tissues.

**Pathway**	**Gene**	**Frequency of Mutations**				**References**
		**HBV-HCC**	**HCV-HCC**	**Alcoholic HCC**	Combined etiologies*	
Telomere maintenance	*TERT*	31%~43%	64%~80%	68%~83%	31%~65%	Nault et al., 2013; Totoki et al., 2014; Fujimoto et al., 2015; Schulze et al., 2015; Kawai-Kitahata et al., 2016; Yang Z. et al., 2016
Wnt/beta-catenin pathway	*CTNNB1*	10%~16%	24%~42%	33%~50%	11%~46%	Satoh et al., 2000; Li et al., 2011; Fujimoto et al., 2012; Guichard et al., 2012; Cleary et al., 2013; Kan et al., 2013; Ahn et al., 2014; Totoki et al., 2014; Schulze et al., 2015; Kawai-Kitahata et al., 2016; Rebouissou et al., 2016; Yao S. et al., 2016
	*AXIN1*	8%			2%~21%	Satoh et al., 2000; Guichard et al., 2012; Kan et al., 2013; Ahn et al., 2014; Totoki et al., 2014; Schulze et al., 2015; Kawai-Kitahata et al., 2016
Cell cycle	*TP53*	32%~68%	12%~23%	25%	13%~52%	Li et al., 2011; Woo et al., 2011; Fujimoto et al., 2012; Guichard et al., 2012; Huang et al., 2012; Cleary et al., 2013; Kan et al., 2013; Nault et al., 2013; Ahn et al., 2014; Meng et al., 2014; Totoki et al., 2014; Schulze et al., 2015; Kawai-Kitahata et al., 2016; Yao S. et al., 2016
	*IRF2*				2%~5%	Guichard et al., 2012; Ahn et al., 2014
	*RB1*	10%			3%~10%	Kan et al., 2013; Ahn et al., 2014; Totoki et al., 2014; Yao S. et al., 2016
	*CCND1*	5%			0%~6%	Ahn et al., 2014; Totoki et al., 2014; Schulze et al., 2015
	*CDKN2A*	5%			2%~8%	Guichard et al., 2012; Ahn et al., 2014; Totoki et al., 2014; Schulze et al., 2015; Kawai-Kitahata et al., 2016
Epigenetic modifier	*ARID1A*	7%			1%~17%	Fujimoto et al., 2012; Guichard et al., 2012; Huang et al., 2012; Cleary et al., 2013; Ahn et al., 2014; Totoki et al., 2014; Schulze et al., 2015; Kawai-Kitahata et al., 2016; Yao S. et al., 2016
	*ARID2*	2%~3%	14%~18%		1%~18%	Li et al., 2011; Fujimoto et al., 2012, 2015; Guichard et al., 2012; Huang et al., 2012; Cleary et al., 2013; Ahn et al., 2014; Totoki et al., 2014; Schulze et al., 2015; Kawai-Kitahata et al., 2016; Yao S. et al., 2016
	*MLL*	4%			2%~7%	Fujimoto et al., 2012; Cleary et al., 2013; Ahn et al., 2014
	*MLL2*	5%			5%~6%	Cleary et al., 2013; Ahn et al., 2014; Schulze et al., 2015
	*MLL3*	8%			1%~7%	Fujimoto et al., 2012; Cleary et al., 2013; Ahn et al., 2014; Schulze et al., 2015
	*MLL4*				3%~7%	Cleary et al., 2013; Schulze et al., 2015
PI3K/Akt/mTOR and Ras/Raf/MAP kinase pathways	*RPS6KA3*	5%			2%~10%	Guichard et al., 2012; Ahn et al., 2014; Totoki et al., 2014; Schulze et al., 2015
	*PIK3CA*				1%~2%	Guichard et al., 2012; Totoki et al., 2014; Schulze et al., 2015; Kawai-Kitahata et al., 2016
	*FGF19*	5%			0%~6%	Ahn et al., 2014; Totoki et al., 2014; Schulze et al., 2015
Stress oxidative pathway	*NFE2L2*				1%~6%	Guichard et al., 2012; Totoki et al., 2014; Schulze et al., 2015; Kawai-Kitahata et al., 2016
	*KEAP1*				2%~8%	Cleary et al., 2013; Totoki et al., 2014; Schulze et al., 2015
JAK/STAT pathway	*JAK1*				1%~9%	Kan et al., 2013; Ahn et al., 2014; Totoki et al., 2014
**Somatic copy number alteration**	**Gene**					
Recurrent focal amplifications	*TERT, FGF19, CCND1, MET, MTDH, BCL9, VEGFA*					Sawey et al., 2011; Guichard et al., 2012; Wang et al., 2013; Totoki et al., 2014; Schulze et al., 2015
Homozygous deletions	*CDKN2A, CDKN2B, CDKN2B, AXIN1, IRF2, ARID1A, RPS6KA3*					Guichard et al., 2012; Wang et al., 2013; Totoki et al., 2014; Schulze et al., 2015

*HBV, hepatitis B virus; HCC, hepatocellular carcinoma; HCV, hepatitis C virus*.

### Recurrent somatic mutations

#### Telomere maintenance

The *TERT* gene encodes a catalytic subunit of telomerase that maintains genomic integrity. *TERT* expression is repressed in somatic cells, but not in proliferative cells in self-renewing tissues and cancers. Somatic mutations in the *TERT* promoter are found across multiple cancer types. Immortality associated with cancer cells has been attributed to telomerase over-expression. *TERT* promoter mutations create a potential binding site for E-twenty-six/ternary complex factors (ETS/TCF) transcription factors and are associated with increased promoter activity, increased expression of *TERT* and increased telomerase activity (Heidenreich et al., 2014; Bell et al., 2015; Borah et al., 2015).

*TERT* promoter mutations are the most frequent somatic genetic alterations observed in HCC, with an overall prevalence of approximately 60%, with ranges from 30 to 40% for HBV-related HCC to 60 to 80% for HCV-related HCC (Nault et al., 2013; Totoki et al., 2014; Fujimoto et al., 2015; Schulze et al., 2015; Kawai-Kitahata et al., 2016; Yang et al., 2016a). HCC with HCV infection and alcohol intake more often harbor TERT promoter mutations than those with HBV infection (Nault et al., 2013). The lower rate of *TERT* promoter mutations in HBV-related HCC could be partially explained by the frequent insertion of HBV DNA in the *TERT* promoter serving as additional mechanism inducing telomerase transcription (Nault et al., 2013).

Somatic *TERT* promoter mutations may represent an early event in liver carcinogenesis in a setting of cirrhosis leading to malignant transformation (Nault et al., 2013, 2014). Mutations in the *TERT* promoter but not in classical liver cancer driver genes such as *CTNNB1* and *TP53* can be found in cirrhotic preneoplasia (Nault et al., 2013). *TERT* promoter mutations have been identified in 6% of low-grade dysplastic nodules, 19% of high-grade dysplastic nodules, 61% of early hepatocellular carcinomas, and 42% of small and progressed HCC, correlating with step-wise development of hepatocarcinogenesis. *TERT* promoter mutations may have utility as a marker for high risk of malignant transformation in cirrhotic tissue (Nault et al., 2014). *TERT* promoter mutations were more frequent in those with lower AFP serum levels, usually in small tumors (Nault et al., 2013; Yang et al., 2016a). Thus, detection of *TERT* promoter mutations may aid in diagnosis of atypical or early HCC cases with lower serum AFP levels.

#### Wnt/β-catenin pathway

Catenin beta 1 (*CTNNB1*), a key signaling transducer in the Wnt pathway, regulates cellular proliferation and differentiation. *CTNNB1* is the most frequently mutated oncogene in HCCs (10–50%) (Satoh et al., 2000; Li et al., 2011; Fujimoto et al., 2012; Guichard et al., 2012; Cleary et al., 2013; Kan et al., 2013; Ahn et al., 2014; Totoki et al., 2014; Schulze et al., 2015; Kawai-Kitahata et al., 2016; Rebouissou et al., 2016; Yao S. et al., 2016). Most mutated residues are in or near phosphorylation sites and prevent phosphorylation-dependent ubiquitination, resulting in abnormal accumulation of β-catenin protein that in turn causes abnormal expression of cell proliferation genes (Klaus and Birchmeier, 2008). Like *TERT* promoter mutations, *CTNNB1* mutation frequency varies in HCC cases by etiological factors, with ranges from 10 to 16% in HBV-related HCC, 20 to 40% in HCV-related HCC, and 30 to 50% in alcoholic HCC (Li et al., 2011; Ahn et al., 2014; Kawai-Kitahata et al., 2016; Rebouissou et al., 2016). *CTNNB1* mutations were associated with lower AFP levels (Rebouissou et al., 2016), indicating detection of *CTNNB1* mutations may also have diagnostic value for HCC with atypical presentation.

*AXIN1* is the second most frequently mutated gene in the Wnt pathway (occurring in 2–20% of HCC cases) (Satoh et al., 2000; Guichard et al., 2012; Kan et al., 2013; Ahn et al., 2014; Totoki et al., 2014; Schulze et al., 2015; Kawai-Kitahata et al., 2016). Axin may be an effective therapeutic target for suppressing growth of HCC tumors (Satoh et al., 2000).

#### p53/cell cycle control pathway

As a tumor suppressor and transcription factor, TP53 can both activate and repress gene expression to initiate cell-cycle arrest, apoptosis, and senescence in response to cellular stresses, including DNA damage, oncogene activation, and hypoxia, to maintain the integrity of the genome (Lee, 2015). *TP53* mutations occurred in approximately 10% to 50% of HCC cases (Li et al., 2011; Woo et al., 2011; Fujimoto et al., 2012; Guichard et al., 2012; Huang et al., 2012; Cleary et al., 2013; Kan et al., 2013; Nault et al., 2013; Ahn et al., 2014; Meng et al., 2014; Totoki et al., 2014; Schulze et al., 2015; Kawai-Kitahata et al., 2016; Yao S. et al., 2016). The mutation spectrum of *TP53* varies depending on etiological and environmental factors. The *TP53* R249S hotspot mutation is particularly associated with aflatoxin B1 exposure, which interacts synergistically with HBV infection to promote hepatocarcinogenesis (Kew, 2003; Hussain et al., 2007). *TP53* mutations occurred more frequently in HBV-related HCC (~30 to 70%) than in non-HBV HCC (Li et al., 2011; Ahn et al., 2014; Kawai-Kitahata et al., 2016). *TP53* mutation is also associated with tumor histological grade. HCCs with a high histological grade have a higher *TP53* mutation rate (40%) than those with a low histological grades (10%) (Ahn et al., 2014).

Abrogation of the *IRF2* (encoding interferon regulatory factor 2), occurring in ~2–5% of HCCs, also leads to impaired TP53 function (Guichard et al., 2012).

Somatic mutations in HCC tumors have also been observed in other cell cycle genes, including the tumor suppressor genes *RB1* (~3–10%) (Kan et al., 2013; Ahn et al., 2014; Totoki et al., 2014; Yao S. et al., 2016) and *CDKN2A* (2~8%) (Guichard et al., 2012; Ahn et al., 2014; Totoki et al., 2014; Schulze et al., 2015; Kawai-Kitahata et al., 2016).

#### Epigenetic modification

Alterations of chromatin regulator genes are recurrently observed in HCC cases. Of 27 HCC tumors with WGS, 52% had somatic mutations or indels in at least one chromatin regulator genes (e.g., *ARID1A, ARID1B, ARID2, MLL*, and *MLL3*; Fujimoto et al., 2012). ARID1A and ARID2 are chromatin remodeling factors, which regulate DNA accessibility to transcription, DNA replication, and repair machineries. *ARID2* mutations were significantly enriched in HCV-associated HCC (18%) compared with HBV-related HCC (2%) (Li et al., 2011). *MLL, MLL2, MLL3*, and *MLL4* genes encode H3K4 methyltransferases that regulate methylation, acetylation and remodeling of nucleosomes. The mutation rates at these genes are presented in (Table 4; Li et al., 2011; Fujimoto et al., 2012, 2015; Guichard et al., 2012; Huang et al., 2012; Cleary et al., 2013; Ahn et al., 2014; Totoki et al., 2014; Schulze et al., 2015; Kawai-Kitahata et al., 2016; Yao S. et al., 2016). Less common mutations (< 10%) were detected in genes involved in PI3K/Akt/mTOR and Ras/Raf/MAP pathways, stress oxidative pathway (Guichard et al., 2012; Cleary et al., 2013; Totoki et al., 2014; Schulze et al., 2015; Kawai-Kitahata et al., 2016) and JAK/STAT pathway (Kan et al., 2013; Nault et al., 2013; Ahn et al., 2014; Totoki et al., 2014) (Table 4).

#### Relationships between mutated genes

Certain subsets of altered genes share the same pathways or interact, contributing to the complexity and heterogeneity of hepatocarcinogenesis. Activated mutations of *CTNNB1* are significantly associated with mutations in the *TERT* promoter (Nault et al., 2013; Totoki et al., 2014). It has been proposed that *TERT* might be a direct target of *CTNNB1* (Hoffmeyer et al., 2012; Zhang et al., 2012). Alterations in *RPS6KA3* are frequently associated with *AXIN1* mutations, suggesting cooperation between RPS6KA3 inactivation and Wnt/β-catenin activation in tumorigenesis (Guichard et al., 2012). On the other hand, a number of mutations appear to be mutually exclusive and rarely appear together in the same tumor, such as *CTNNB1* mutations with *TP53* mutations (Guichard et al., 2012; Ahn et al., 2014) and *AXIN1* mutations with *CTNNB1* mutations (Satoh et al., 2000). *ARID1A*/*ARID2* mutations are negatively associated with mutations in *TP53* (Lee, 2015). The consequences of network interactions between driver mutations may offer deeper insight into tumorigenesis.

## HCC genetics and precision medicine

### Somatic mutations and therapeutic targets of HCC

Targeted therapy based on genomic alterations is a core tenet of precision treatment. Exome sequencing analysis of 243 liver tumors found that 28% of patients harbor at least one alteration potentially targetable by an FDA-approved drug, and 86% harbored a mutation targetable by a drug studied in phase I to phase III clinical trials (Schulze et al., 2015). β-catenin reduction in *CTNNB1*-mutated HCCs in a murine model led to complete tumor response, showing a clear benefit of therapeutic targeting of this molecule (Delgado et al., 2015). Adenovirus mediated gene transfer of wild-type *AXIN1* induced apoptosis in hepatocellular cancer cells that had accumulated β-catenin as a consequence of mutations in *APC, CTNNB1*, or *AXIN1* genes, suggesting that AXIN1 may be an effective therapeutic molecule for suppressing HCC growth (Satoh et al., 2000).

### Somatic mutations and HCC prognosis

Somatic mutations of driver genes may be predictive of HCC prognosis. In a study of over 300 HCC Chinese patients, *TP53* hotspot mutations (R249S and V157F were strongly associated with decreased overall survival, indicating that these mutations can be used as prognostic markers in HCC in patients at risk for high aflatoxin exposures (Woo et al., 2011). Mechanistically, a synergistic interaction of aflatoxin B1 induced *TP53* mutations together with HBV chronic inflammation may advance the development of HCC. In a multivariate analysis of 231 Korean HCC patients, the *RB1* somatic mutation was the only independent prognostic factor for reduced cancer-specific survival and accelerated recurrence (Ahn et al., 2014). In a cohort of resected HCCs, *CDKN2A* inactivation was associated with poor prognosis (Schulze et al., 2015). These predictive and prognostic molecular markers may have clinical utility for personalized treatment plans.

## Conclusion, challenges and future directions

The development of HCC is multifactorial with viral, host and environmental factors contributing to chronic inflammation, cirrhosis and ultimately, hepatocarcinogenesis (Figure 1). Interplay between host germline variants, persistent high HBV viral load, viral genotypes and mutations, HBV integration into host chromosomes, the oncogenic potential of the HBx protein, and the occurrence of somatic cancer driver mutations may contribute independently or jointly to the oncogenesis of HBV-related HCC (Figure 1). We hope that eventually genetic profiling of the virus and host will identify the individuals who are at higher risk of HCC and those who will benefit most from HCC treatment options. Since early HCC is largely asymptomatic and biopsies are rarely performed; there is a paucity of pre-tumor and early-stage tumor tissue available, a major impediment for genetic interrogation. This is in contrast to breast, colon, and prostate cancers where biopsies are standard of care for patients with abnormal findings during routine screening. Validated genetic biomarkers would have utility for early diagnosis, molecular classification of tumors, and prognostics as well as identify new targets for drug interventions. With the advent of ever larger HCC cohorts, denser, population-specific genotyping arrays, and next generation whole exome/genome sequencing of HCC family clusters, it may be possible to provide earlier diagnosis of HCC and to develop bespoke treatment for persons with HCC to improve outcomes.

## Author contributions

PA and CW: conceived and wrote the paper; JX: wrote and revision of the manuscript; YY: revision of the manuscript.

### Conflict of interest statement

The authors declare that the research was conducted in the absence of any commercial or financial relationships that could be construed as a potential conflict of interest.
